# A machine-learning clustering approach for reference interval estimation of liver enzymes from hospital laboratory big-data

**DOI:** 10.6026/973206300211069

**Published:** 2025-05-31

**Authors:** Prakruti Dash, Saurav Nayak

**Affiliations:** 1 Department of Biochemistry, AIIMS, Bhubaneswar, India; 2 Department of Biochemistry, IMS & Sum Hospital Campus 2, Phulnakhara, Bhubaneswar, Odisha, India

**Keywords:** Reference interval, clustering, machine learning

## Abstract

It is of interest to establish clinically valid reference intervals (RIs) for the liver enzymes aspartate transaminase (AST) and Alanine aminotransferase (ALT) using a combination of unsupervised machine learning clustering and robust outlier detection applied to real-world laboratory big data. Four outlier detection methods were each combined with four clustering algorithms to identify homogeneous subgroups and the largest cluster from each combination was used to estimate RIs based on percentile cut-offs. Among the tested combinations, DBSCAN with Tukey's fences or Local Outlier Factor achieved optimal performance, covering 100% of the validation data. The widest intervals were derived using Local Outlier Factor, while Isolation Forest yielded the narrowest. Ultimately, the study estimated the reference intervals for AST and ALT to be 15-41 U/L and 11-46 U/L, respectively.

## Background:

The reference interval for multiple diagnostic tests is delineated by threshold values within which the test results of a designated percentage (often 95%) of ostensibly healthy individuals are expected to remain. The threshold or limiting values for the reference interval typically correspond to the 0.025 and 0.975 quartiles of the test result distribution within the reference population [1]. Reference intervals (RIs) are obtained from reference distributions, often representing a 95% confidence interval and characterize a particular population. The computation of RIs encompasses parametric and nonparametric methodologies, identification of outliers, segmentation and confidence intervals. Five percent of all findings from healthy individuals will fall beyond the reported reference interval and will thus be designated as 'abnormal.' Reference persons delineate the classical cascade, a reference sample group, reference values, reference distribution, reference boundaries and reference intervals (RIs) [2]. The reference individuals constitute the reference sample group for assessing the values of the reference population. RIs should ideally be established based on a healthy population utilizing direct methods [3]. Health, however, is a subjective state devoid of a universal definition. The assessment of good health and the establishment of normality for a reference individual may encompass many evaluations, including medical history, physical examination and/or specific clinical laboratory testing [4]. To ensure repeatability and standardization, the pre-analytical and analytical components must be precisely specified and articulated, as the pre-analytical phase is recognized to exhibit the highest incidence of mistakes [5]. Nonetheless, indirect methods, commonly referred to as data mining derived from prior laboratory data, can also prove beneficial. Diverse techniques can be employed to identify a cohort of healthy individuals from a general hospital population and reference values are derived from hospital data utilizing statistical methods, including Bhattacharya analysis and its variants [6, 7]. Data mining utilizes the existing dataset derived from patient outcomes. In recent years, the emergence of the digital era has heightened interest in empirical investigations to obtain reference intervals from mixed datasets of physiological and pathological test outcomes [8, 9, 10- 11]. Real-world data refers to information gathered from genuine, routine clinical occurrences and interactions, in contrast to data produced in regulated or experimental environments. This advancement substantially lowers the economic burden of establishing RIs and resolves the issue of applying RIs from alternative sources to diverse testing systems and unique populations [12, 13]. Alanine aminotransferase (ALT) and aspartate transaminase (AST) are important parameters of liver function tests and play a crucial role in a multitude of diagnoses, treatments and prognoses of diseases. The use of proper RIs for clinical diagnosis, therapy and management is crucial [14]. The serum levels of ALT and AST are frequently measured in patients with suspected or proven liver diseases, as the serum levels of ALT and AST are considered surrogate markers of liver injury [15, 16]. The upper limit of normal aminotransferases is taken as 40 U/L and a clinical diagnosis is considered when enzymes double or triple in value. However, recently, research groups argued that the genuine normal values for serum ALT & AST levels are far lower than the widely accepted range of normal values [17, 18-19]. Therefore, it is of interest to utilize Machine Learning (ML) Unsupervised Clustering methods combined with Outlier Detectors to determine an RI for AST and ALT from Laboratory Patient Big Data.

## Methodology:

## Study setting:

The study was conducted in the Department of Biochemistry at a Tertiary Care Hospital in Eastern India. The ethical permission of the study was obtained from the Institutional Ethical Committee vide Ref No. T/IM-NF/Biochem/22/181. Data was collected from Laboratory e-records based on CDAC eSushrut Laboratory Information System by retrieving data retrospectively in a time period of 1 year of 2023-24.

## Study criteria:

As the population defined for the study was hospital-based, stringent inclusion and exclusion criteria were set so as to successfully cordon off data that might be affected by the patient's condition. The inclusion criteria to be considered a datapoint was defined as any adult patient between 18-60 years of age who had Sex, Total Bilirubin (TBil), AST, ALT and Diagnosis fields non-blank. All patients admitted to the In-Patient Department (IPD) were excluded. Patients with TBil greater than 1 mg/dL were excluded. All obstetrics patients were excluded, as well as patients of the Department of Surgery, Gastroenterology, Gastro Surgery, Oncology, Haemato-Oncology, Radiation Oncology, Urology, Nephrology, Trauma and Emergency and Burns. Any diagnosis, including but not limited to jaundice, chest pain, stomachache, fever, *etc.*, which might have direct or indirect adverse effects on liver health, was excluded.

## Dataset split:

The dataset was split randomly into two segments: Learning and validation. In the learning dataset comprising 70% of the total data, the outlier detection and clustering algorithms were applied and the reference interval's lower and upper limit was generated. The validation dataset (30%) was utilized to validate the reference interval generated.

## Outlier detection:

Outlier Detection was carried out by 4 methods. Tukey's Fences (TF) was the conventional method applied which multiplies 1.5 with the Interquartile Range and takes that as the maximum deviation from the 1st and 3rd Quartile (Q1 - Q3) as the range. Any data point out of it was considered an outlier. Local Outlier Factor (LOF) is a density-based method that flags apparently unusual data points based on how isolated they are from their immediate neighbors. It compares the closeness of each point with that of surrounding points. If a point is much less dense than its neighbors, it is likely an outlier. This is especially useful in medical data when detecting patients whose laboratory results deviate notably from the norm within a small group. Isolation Forest (IF) takes a different approach by randomly splitting the data and seeing how quickly it isolates certain points. Outliers are typically separated from the majority of data more quickly, *i.e.*, fewer "splits" in this tree-based algorithm. The Elliptical Envelope (EE) fits an imaginary statistical ellipse around what it considers normal data points, assuming the data roughly follow a Gaussian (bell-shaped) pattern. Points that lie too far outside this ellipse are flagged as outliers.

## Estimation of reference range:

Each of the outlier detection methods was paired with a machine learning unsupervised clustering algorithm to estimate the reference range. Density-based spatial clustering of applications with noise (DBSCAN) groups together points that are closely packed and marks points that lie alone in low-density regions as outliers. It requires two main parameters: the maximum distance between neighbouring points (epsilon) and the minimum number of points needed to form a dense region. Balanced iterative reducing and clustering using hierarchies (BIRCH) is designed for large datasets, incrementally building a tree structure (called a CF tree) that grows as new data arrives. It clusters data by hierarchically merging similar points, allowing for rapid, memory-efficient processing. Outliers can surface as points or small clusters that do not merge well with existing larger clusters. Hierarchical clustering (HC) begins by treating each data point as its own cluster and then iteratively merges clusters based on a chosen distance metric. The outcome is often shown as a dendrogram, which visually depicts how clusters join at different levels. Anomalies may be identifiable as points or small groups that remain separate until very late in the merging process. Agglomerative clustering (AC) is a specific "bottom-up" form of hierarchical clustering. It starts by placing each data point in its own cluster and merges them step by step based on similarity. Outliers can be those entities that fail to merge with others at early stages and only fuse with the main cluster at a much higher distance threshold. In each clustering modality, the largest individual cluster was selected and 5 intervals in ascending order of stringency, *i.e.*, Minimum (Min) - Maximum (Max), Q1-Q3, 0.05th - 99.95th percentile, 0.1 - 99.9th percentile, 1st - 99th percentile and 5th - 95th percentile. Each reference interval was compared to a combination of Tukey's fences with a 2.5th-97.5th percentile method based on EP28-A3c. The percentage of the validation dataset that fits in the above-mentioned reference intervals will be considered for determination.

## Results:

A total of 9519 data points were included in this study. The median age was 42 (30 - 51) years. Approximately 45% were males and 55% were females. The values were similar in both the learning and validation datasets (Table 1). After being subjected to iterative removal of outliers till no outliers were left, IF was the method with the highest removals, whereas LOF was the least. The distribution of AST and ALT values, however, remained fairly similar in all cases datasets. After outlier removal, each dataset was administered a Machine Learning Unsupervised Clustering Algorithm. These algorithms broke down the datasets into multiple clusters. Amongst these, the single largest individual cluster was selected as the eminent set of data points for reference interval estimation. Different cut-off limits were also evaluated based on percentile scores of the data points in the largest individual cluster. Based on these permutations, the values for the probable cut-offs for lower and higher values of the reference interval were determined based on the percentile value of the largest clusters datasets (Table 2). This showed that in all the outlier mechanisms, DBSCAN and AC held on to most data points as the single largest cluster, while the other two methods scattered the data, thus having a small relative size. DBSCAN had the largest relative size in each case. As per the existing guidelines, the Percentile Method was used with the outliers removed dataset. For AST as well as ALT, the widest range was with LOF (13 - 44 and 10 - 51, respectively). If was the narrowest in both the analyses (18 - 31 for AST and 13 - 30 for ALT). The validation dataset was applied to all the method combinations to estimate the coverage based on the percentiles. This has been summarized in Table 3. The coverage estimation depicts the utility of DBSCAN when used along with TF or LOF, as it shows 100% coverage of validation data in line with the percentile method. Across the board, HC is the worst-performing. Also, by utilizing DBSCAN with TF or LOF, the more stringent criteria of the 5th - 95th percentile can be applied to the dataset to provide a robust and encompassing method combination to determine lower and upper cut-off points for reference interval. The strictest method of using quartiles, however, leaves many significant data points outside the reference, thus proving ineffective. As DBSCAN, along with LOF, had the largest relative size, it may be considered the most suitable method for the determination of reference interval. The reference interval thus estimated is 15-41 for AST and 11 - 46 for ALT.

## Discussion:

The primary objective of this study was to determine the essential role of laboratory-based patient data in quantifying the Reference Interval from apparently healthy patients. The exclusion criteria were made robust so as to prevent overlapping of any patient who would actually be dealing with raised hepatic enzyme levels. This, in turn, warrants the utilization of unsupervised machine learning models. These models work unbiasedly in outlier-removed databases to bring together similar values, the primary goal of a population-based reference range. Velev *et al.* provide a very similar experience in a Puerto Rican population concerned with Chronic Kidney Disease. The task at hand was to define what is actually healthy in a population being studied. They reach the conclusion that it is better to define healthy as being within 95% of the mean of the distribution of individuals of the same gender and age [20]. In our study, the primary focus of data processing also relied on this "definition." A disease-free condition is different from an individual with proper liver enzyme status. Any factor that can remotely affect this status - directly or indirectly - formed the basis of the stringent exclusion criteria [21]. AST and ALT form an integral part of the Liver Function Test (LFT) in routine biochemistry laboratory; however, most disease conditions do not have any connotations with these unless it has some incremental effect on hepatic or cardiac function or any pathological morphology of the patient. The exclusion of most, if not all, such affected individuals forms a dataset that is, although laboratory and hospital data-based, at par with conventions and norms being practiced [19].

Lidbury *et al.* examined the statistical significance of laboratory test panels, specifically concentrating on liver function tests. Utilizing other elements of the liver function panel, they successfully predicted the normality or abnormality of Y-glutamyl transferase (GGT) with an accuracy of 90% through a tree-based machine learning model [22]. They determined that GGT provided minimal supplementary value beyond the other elements of a standard liver function panel. A notable example of this work is the research conducted by Azarkhish *et al.* wherein a neural network model forecasted iron deficiency anemia and serum iron levels utilizing features derived from a standard complete blood count [23]. Luo *et al.* presented a machine learning algorithm that accurately predicted abnormal serum ferritin levels, achieving 97% accuracy by employing a random forest imputation method to address missing laboratory features, which were subsequently input into a logistic regression model [24]. Observing similar studies, Rai et al in a pilot work in a tertiary care setting reported serum AST to be 11 to 43 IU/L in males and 10.7 to 37.2 IU/L in females, and for ALT a general reference range of 4.6 to 47 IU/L [25]. This is in line with our study. When a validating cohort of the same derived population was analyzed for coverage, it was well noticed that the narrowest band of reference range derived from the clustering fits an equivalent number of the dataset as the conventional method does. Thus, the efficacy of laboratory-data-based RI through unsupervised clustering is implied. However, there have been certain limitations to the study, as a longer period would have provided a larger dataset to analyze. Secondly, the data is representative of a narrow band of the true population as our institution is a tertiary care center. Finally, the methodology couldn't be modified to encompass subgrouping based on gender and age.

## Conclusion:

Our study provides an estimated RI for AST and ALT in lines of conventional methodology, derived solely from the apparently unaffected liver enzyme population from the laboratory e-records. The reference interval thus estimated is 15-41 U/L for AST and 11 - 46 U/L for ALT. Similar studies can be conducted for all routine biochemistry parameters, with stringent measures, outlier detection and robust clustering models.

## Declaration on publication ethics:

The author's state that they adhere with COPE guidelines on publishing ethics as described elsewhere at https://publicationethics.org/. The authors also undertake that they are not associated with any other third party (governmental or non-governmental agencies) linking with any form of unethical issues connecting to this publication. The authors also declare that they are not withholding any information that is misleading to the publisher in regard to this article.

## License statement:

This is an Open Access article which permits unrestricted use, distribution and reproduction in any medium, provided the original work is properly credited. This is distributed under the terms of the Creative Commons Attribution License.

## Figures and Tables

**Table 1:** Distribution of AST and ALT after outlier removal by various methods

**Method**	**AST**					**ALT**				
	**Final N**	**Removed (%)**	**Min**	**Max**	**Median (Q1-Q3)**	**Final N**	**Removed (%)**	**Min**	**Max**	**Median (Q1-Q3)**
TF	8018	15.8	5	45	24 (20 - 30)	8048	15.5	4	51	22 (16 - 30)
LOF	8165	14.2	10	51	25 (20 - 30)	8182	14	6	56	22 (16 - 30)
IF	5402	43.3	18	31	24 (21 - 27)	5420	43.1	13	30	20 (17 - 25)
EE	7368	22.6	12	37	24 (20 - 29)	7420	22.1	4	40	21 (16 - 27)
Final N = No of datapoints left after
Outlier removal, Actual N = 9519

**Table 2:** Data distribution of largest cluster by each method combination

**Parameter**	**OR - CA**	**Size**	**RS (%)**	**Min**	**Max**	**0.05**	**0.01**	**1**	**5**	**25**	**50**	**75**	**95**	**99**	**99.9**	**99.95**
ST	TF - DBSCAN	5613	84.2	5	45	7	9	11	15	20	24	30	39	44	45	45
	TF - BIRCH	987	14.8	20	22	20	20	20	20	20	21	22	22	22	22	22
	TF - HC	336	5	22	22	22	22	22	22	22	22	22	22	22	22	22
	TF - AC	4142	62.2	5	29	6	8	11	14	19	22	25	29	29	29	29
	LOF - DBSCAN	5682	85.3	10	48	10	10	12	15	20	25	30	41	46	48	48
	LOF - BIRCH	987	14.8	20	22	20	20	20	20	20	21	22	22	22	22	22
	LOF - HC	336	5	22	22	22	22	22	22	22	22	22	22	22	22	22
	LOF - AC	2893	43.4	25	51	25	25	25	25	27	30	35	44	50	51	51
	IF - DBSCAN	3782	56.8	18	31	18	18	18	18	21	24	27	30	31	31	31
	IF - BIRCH	987	14.8	20	22	20	20	20	20	20	21	22	22	22	22	22
	IF - HC	336	5	22	22	22	22	22	22	22	22	22	22	22	22	22
	IF - AC	1978	29.7	24	31	24	24	24	24	25	27	29	31	31	31	31
	EE - DBSCAN	5158	77.4	12	37	12	12	13	15	20	24	29	35	37	37	37
	EE - BIRCH	987	14.8	20	22	20	20	20	20	20	21	22	22	22	22	22
	EE - HC	336	5	22	22	22	22	22	22	22	22	22	22	22	22	22
	EE - AC	3325	49.9	22	37	22	22	22	22	24	27	31	36	37	37	37
ALT	TF - DBSCAN	5633	84.5	4	51	6	7	9	11	16	22	30	44	50	51	51
	TF - BIRCH	738	11.1	16	18	16	16	16	16	16	17	18	18	18	18	18
	TF - HC	258	3.9	17	17	17	17	17	17	17	17	17	17	17	17	17
	TF - AC	4332	65	4	30	6	6	8	10	15	19	24	29	30	30	30
	LOF - DBSCAN	5724	85.9	8	56	8	8	9	11	16	22	30	46	54	56	56
	LOF - BIRCH	738	11.1	16	18	16	16	16	16	16	17	18	18	18	18	18
	LOF - HC	258	3.9	17	17	17	17	17	17	17	17	17	17	17	17	17
	LOF - AC	4765	71.5	6	34	7	8	9	11	15	20	26	32	34	34	34
	IF - DBSCAN	3793	56.9	13	30	13	13	13	13	17	20	25	29	30	30	30
	IF - BIRCH	738	11.1	16	18	16	16	16	16	16	17	18	18	18	18	18
	IF - HC	258	3.9	17	17	17	17	17	17	17	17	17	17	17	17	17
	IF - AC	2613	39.2	13	23	13	13	13	13	15	18	21	23	23	23	23
	EE - DBSCAN	5193	77.9	4	40	6	7	9	11	16	21	27	36	40	40	40
	EE - BIRCH	738	11.1	16	18	16	16	16	16	16	17	18	18	18	18	18
	EE - HC	258	3.9	17	17	17	17	17	17	17	17	17	17	17	17	17
	EE - AC	3152	47.3	4	23	5	6	8	10	14	17	20	23	23	23	23
OR-CA: Outlier Removal and Clustering Algorithm Combination.
RS: Relative Size of Largest Cluster to the Learning Dataset. %ile: Percentile.
Min: Minimum value. Max: Maximum value

**Table 3:** Percentage coverage of validation dataset by various method combinations for AST and ALT

**Analyte**	**OR - CA**	**Q1-Q3**	**5-95th %ile**	**1-99th %ile**	**0.1-99.9th %ile**	**0.05-99.5th %ile**	**Min-Max**	2.5-97.5th %ile *
AST	TF - %ile							100
	TF - DBSCAN	68.7	100	100	100	100	100	
	TF - BIRCH	15.5	15.5	15.5	15.5	15.5	15.5	
	TF - HC*	0	0	0	0	0	0	
	TF - AC	41.3	80	80	80	80	80	
	LOF - %ile							100
	LOF - DBSCAN	68.7	100	100	100	100	100	
	LOF - BIRCH	15.5	15.5	15.5	15.5	15.5	15.5	
	LOF - HC*	0	0	0	0	0	0	
	LOF - AC	29.7	47.6	47.6	47.6	47.6	47.6	
	IF - %ile							77.6
	IF - DBSCAN	45	74.9	77.6	77.6	77.6	77.6	
	IF - BIRCH	15.5	15.5	15.5	15.5	15.5	15.5	
	IF - HC*	0	0	0	0	0	0	
	IF - AC	27.6	41.5	41.5	41.5	41.5	41.5	
	EE - %ile							100
	EE - DBSCAN	65.7	97	100	100	100	100	
	EE - BIRCH	15.5	15.5	15.5	15.5	15.5	15.5	
	EE - HC*	0	0	0	0	0	0	
	EE - AC	41.5	70.2	70.2	70.2	70.2	70.2	
ALT	TF - %ile							100
	TF - DBSCAN	70.7	100	100	100	100	100	
	TF - BIRCH	14.1	14.1	14.1	14.1	14.1	14.1	
	TF - HC	6.3	6.3	6.3	6.3	6.3	6.3	
	TF - AC	49.2	76.5	78.7	78.7	78.7	78.7	
	LOF - %ile							100
	LOF - DBSCAN	70.7	100	100	100	100	100	
	LOF - BIRCH	14.1	14.1	14.1	14.1	14.1	14.1	
	LOF - HC	6.3	6.3	6.3	6.3	6.3	6.3	
	LOF - AC	58.1	82.2	86.1	86.1	86.1	86.1	
	IF - %ile							75.8
	IF - DBSCAN	49.6	73.6	75.8	75.8	75.8	75.8	
	IF - BIRCH	14.1	14.1	14.1	14.1	14.1	14.1	
	IF - HC	6.3	6.3	6.3	6.3	6.3	6.3	
	IF - AC	33.4	47.4	47.4	47.4	47.4	47.4	
	EE - %ile							93.1
	EE - DBSCAN	60.6	89.8	96.7	96.7	96.7	96.7	
	EE - BIRCH	14.1	14.1	14.1	14.1	14.1	14.1	
	EE - HC	6.3	6.3	6.3	6.3	6.3	6.3	
	EE - AC	41.5	70.2	70.2	70.2	70.2	70.2	
OR-CA: Outlier Removal and Clustering Algorithm Combination.
*: Only for Percentile method. %ile: Percentile. Q1: 1st Quartile, Q3: 3rd Quartile.
*: HC based Clustering was unsuccessful in generating meaningful cluster for AST
